# The Correlation Between the Lactate/Albumin Ratio and Sequential Organ Failure Assessment (SOFA) Score in Patients With Sepsis and Septic Shock

**DOI:** 10.7759/cureus.65616

**Published:** 2024-07-29

**Authors:** Madhulika L Mahashabde, Yash R Bhimani, Harin M Bhavsar

**Affiliations:** 1 General Medicine, Dr. D. Y. Patil Medical College, Hospital and Research Centre, Dr. D. Y. Patil Vidyapeeth, Pune, IND

**Keywords:** icu, septic shock, sepsis, sofa score, lactate albumin ratio

## Abstract

Background

Sepsis is defined as a life-threatening organ dysfunction caused by a dysregulated host response to infection, often resulting in severe outcomes such as septic shock and death. Globally, sepsis ranks among the most common causes of illness and death. The Sequential Organ Failure Assessment (SOFA) score is an established marker used to assess and predict the extent of organ failure in septic patients. The introduction of novel markers, such as the lactate/albumin (L/A) ratio, serves as a prognostic indicator in critical care settings, particularly for patients with sepsis. In this context, a higher L/A ratio upon admission aids in assessing disease severity and improving clinical decision-making to reduce mortality and adverse outcomes, which we aim to correlate through our study.

Materials and methods

This was an observational cross-sectional analysis conducted on 100 patients aged over 18 years who met the “Sepsis-3” guidelines and were admitted to the medical intensive care unit of Dr. D. Y. Patil Hospital, Pune, Maharashtra, India, between October 2022 and May 2024. Patients with chronic liver disease classified as Child-Pugh class C were excluded, as were those with chronic kidney disease (CKD). Written informed consent was obtained from each participant before the study. Data were collected through physical examination, routine laboratory investigations, and radiological assessments. Statistical analysis was performed using IBM SPSS version 20 (IBM Corp., Armonk, NY). Descriptive statistical analyses were conducted using the SPSS data editor. Statistical significance was considered at a p-value of less than 0.05 for all analyses.

Results

In the study population, 78 patients survived, while 22 patients died. The L/A ratio and SOFA score were significantly higher in non-survivors compared to survivors, both upon admission and thereafter, with statistical significance (p < 0.05). The correlation between the L/A ratio and the SOFA score was examined upon admission at 24 hours, 48 hours, day 7, and day 28. Pearson correlation analysis revealed statistically significant results (p < 0.05) throughout the entire study period.

Conclusion

A high L/A ratio, along with the SOFA score at ICU admission, was associated with a grave prognosis and poor outcomes, serving as independent risk factors for ICU admission. Therefore, patients with a high L/A ratio and SOFA score should be identified early and managed aggressively to avoid poor outcomes. Our study demonstrates that combining serum lactate and serum albumin levels into the L/A ratio significantly enhances prognostic accuracy compared to using serum lactate alone.

## Introduction

Sepsis is more of a syndrome than a single illness, resulting from intricate interactions between infectious pathogens and the immune system. Normally, the immune system controls infections effectively, but in sepsis, this response becomes uncontrolled, leading to widespread inflammation and potential multi-organ failure [1]. Sprung et al. examined the incidence and mortality rates associated with sepsis as early as 1990 among critically ill patients, emphasizing the need for early detection and treatment [2].

The pathophysiology of sepsis is multifaceted, involving pro-inflammatory mediators such as tumor necrosis factor (TNF), interleukin-1 (IL-1), and interleukin-6 (IL-6), which are produced by the body in response to infection. These cytokines activate various cells and coagulation pathways, leading to widespread inflammation and coagulation abnormalities [3]. The combined effects of inflammation, endothelial dysfunction, coagulation abnormalities, immune suppression, and metabolic dysregulation result in multi-organ failure, which is the primary cause of mortality in sepsis. Commonly affected organs include the kidneys, leading to acute renal injury, and the lungs, resulting in acute respiratory distress syndrome. Other affected organs often include the liver and the cardiovascular system [4,5]. Common signs and symptoms of sepsis include fever with or without chills, hypothermia, rapid heart rate, hypotension, rapid respiratory rate, altered mental status, and decreased urine output. These symptoms arise from the systemic inflammatory response and subsequent organ dysfunction [6]. A timely and accurate diagnosis of sepsis is essential for effective management.

The Sequential Organ Failure Assessment (SOFA) score is commonly used to assess the severity of organ dysfunction in sepsis patients, with higher scores correlating with increased mortality rates [7]. Elevated lactate levels indicate anaerobic metabolism due to inadequate tissue perfusion. In sepsis, cellular hypoxia and mitochondrial dysfunction lead to increased lactate production. Serum albumin levels can decrease in critically ill patients due to inflammation, fluid shifts, and liver dysfunction. Elevated lactate levels indicate hypoperfusion and stress, while albumin levels can reflect the patient's overall physiological state and nutritional status. Therefore, the lactate/albumin (L/A) ratio can provide additional prognostic information beyond that of lactate alone, particularly in predicting in-hospital mortality. The L/A ratio is increasingly recognized as a robust predictor of in-hospital mortality across various medical conditions, highlighting its potential for early identification of high-risk patients [8].

Aims and objectives

This research aims to study the correlation between the L/A ratio and SOFA score in patients with sepsis and septic shock for early detection and prompt management. It also aims to assess whether the L/A ratio is a more effective indicator for sepsis patients than traditional serum lactate levels.

## Materials and methods

Study design and setting

This observational, cross-sectional analysis was conducted on 100 patients admitted to the medical intensive care unit of Dr. D. Y. Patil Hospital, Pune, Maharashtra. The study took place from October 2022 to May 2024, during which each patient underwent thorough clinical examinations and investigations. The study received approval from the Institutional Ethics Committee of DYPMCH (approval number: IESC/PGS/2022/06, dated September 28, 2022).

Inclusion criteria

The study included patients older than 18 years who were admitted to the Medical Intensive Care Unit and met the criteria of the Third International Consensus Definitions for Sepsis and Septic Shock (Sepsis-3).

Exclusion criteria

All cases of chronic liver disease with Child-Pugh class C and patients with chronic kidney disease (CKD) were excluded from the study.

Sample size

One hundred patients who satisfied the “Sepsis-3” guidelines were selected. The prevalence of sepsis in India is 28.3%, as reported in the reference study “Epidemiology of Adult-population Sepsis in India: A Single Center 5 Year Experience” conducted by Chatterjee et al. (2017) [9]. With a 95% confidence interval and an acceptable difference of 9%, the minimum sample size calculated was 97 using WinPepi 11.38 software (Joseph H. Abramson, Jerusalem, Israel).

Data and sample collection

Our study was conducted at Dr. D. Y. Patil Hospital and Research Centre, Pimpri, Pune, with approval from the ethical committee. We selected 100 patients admitted to the medical ICU who satisfied the “Sepsis-3” guidelines. Each patient was thoroughly informed about the study, and written informed consent was obtained before participation. A detailed clinical history was collected, and comprehensive physical and radiological examinations were performed. Routine laboratory investigations were sent along with sepsis markers such as serum lactate, serum procalcitonin, D-dimer, and fibrinogen. Hemogram analyses were conducted using the DxH 900 analyzer (Beckman Coulter, Brea, California), based on the Coulter Principle, which directly counts and sizes cell volume by detecting changes in electrical resistance as particles pass through a small aperture. Renal function tests (RFTs) and liver function tests (LFTs), as well as serum electrolytes, were analyzed using the ARCHITECT c8000 (Abbott, Green Oaks, Illinois) employing spectrophotometric measurement methods. Serum samples were centrifuged at 4,000 rpm for 20 minutes and excluded if found to be lysed or lipemic. The sample collection procedure began by identifying and explaining it to the patient. Supplies were gathered, hand hygiene was performed, and gloves were worn. The patient was positioned, a tourniquet was applied, and a venipuncture site was selected. The site was cleaned with an antiseptic and allowed to dry. The needle and holder were assembled, and the needle was inserted into the vein. Blood was collected in an ethylenediaminetetraacetic acid (EDTA) (lavender) vacutainer for CBC and in a plain (red) vacutainer for the dengue profile, LFT, RFT, and serum electrolytes, following the correct order of draw to avoid contamination. The tourniquet was released, the needle was withdrawn, and pressure was applied to the site. The needle was disposed of in a sharps container, the tubes were labeled, and the puncture site was bandaged. The patient's well-being was checked, samples were transported and stored as required, and the procedure was documented in the patient’s record.

Serum lactate was measured using the enzymatic method with the help of the Dimension EXL 200 analyzer. Serum albumin levels were determined using the bromocresol purple method with the Architect CI 2000 SR analyzer. The L/A ratio was calculated by dividing serum lactate levels by serum albumin levels. The SOFA score was computed using a web-based calculator available at https://intensivecarenetwork.com/Calculators/Files/Sofa.html.

Consent

Written informed consent was obtained from each patient. The study recruited 100 patients, explained the procedure and purpose to them, and obtained their informed consent.

Statistical analysis

Analysis was performed using IBM SPSS Statistics Version 20 (Statistical Package for the Social Sciences, Released 2011; IBM Corp., Armonk, NY). Descriptive statistical analysis, including computation of the mean and standard deviation, was conducted using SPSS software. Statistical significance for parametric and nonparametric data, including p-values, was calculated using Pearson's correlation and the chi-square test. A significance level of less than 0.05 was used for all analyses.

## Results

The analysis of baseline qualitative characteristics in 100 patients with sepsis and septic shock reveals that the majority of patients were over 60 years old (43%), followed by those aged 41-60 years (37%), and a smaller proportion aged 18-40 years (20%). The sample had a higher prevalence of males (65%) compared to females (35%). Among the systems involved, the respiratory system was most frequently affected (30%), followed by renal (20%), gastroenterology (16%), and other systems (34%). Regarding outcomes, a substantial majority of patients survived (78%), while 22% succumbed to the condition, as shown in Table 1. Analysis of vitals at presentation in patients with sepsis and septic shock is shown in Table 2.

**Table 1:** Analysis of baseline qualitative characteristics in patients with sepsis and septic shock (N = 100)

Parameter	Frequency (percentage)
Age (years)	
18-40	20 (20)
41-60	37 (37)
>60	43 (43)
Sex	
Male	65 (65)
Female	35 (35)
System involved	
Respiratory	30 (30)
Gastrointestinal tract	16 (16)
Renal	20 (20)
Others	34 (34)
Outcome	
Survived	78 (78)
Died	22 (22)

**Table 2:** Analysis of vitals at presentation in patients with sepsis and septic shock (N = 100) DBP: diastolic blood pressure; GCS: Glasgow Coma Scale; RR: respiratory rate; SBP: systolic blood pressure

Parameter	Mean	Median	SD	Minimum	Maximum
Pulse	83.8	84.0	17.5	50.0	160.0
SBP	113.0	110.0	15.8	80.0	160.0
DBP	72.4	70.0	11.8	50.0	110.0
RR	25.89	26.00	7.847	14	50
GCS	12.4	13.0	2.1	5.0	15.0

Comparison of serum lactate between survivors and non-survivors

Table 3 compares the mean serum lactate levels at different time points (0 hours, 24 hours, 48 hours, day 7, and day 28) between survivors and non-survivors. At all measured times, non-survivors had higher mean lactate levels compared to survivors. However, none of the differences reached statistical significance (p > 0.05).

**Table 3:** Association of serum lactate between survivors and non-survivors (N = 100) The association was carried out using the Pearson chi-square test, and the p-value was significant at less than 0.05.

Parameter	Outcome (mean ± SD)		p-value
	Survivors (N = 78)	Non-survivors (N = 22)	
Serum lactate (0 hour)	1.18 ± 0.81	4.19 ± 1.75	0.200
Serum lactate (24 hours)	1.23 ± 0.92	2.32 ± 1.42	0.478
Serum lactate (48 hours)	1.12 ± 0.72	2.48 ± 1.61	0.243
Serum lactate (day 7)	1.04 ± 0.58	3.09 ± 1.41	0.166
Serum lactate (day 28)	0.78 ± 0.39	4.49 ± 0.49	0.395

Comparison of L/A ratio between survivors and non-survivors

In the study population, the mean L/A ratio at admission was 0.36 in survivors and 1.36 in non-survivors, indicating a significant difference with a notably higher mean L/A ratio in the mortality group. Table 4 demonstrates that the difference in the mean L/A ratio between survivors and non-survivors was statistically significant at all points during the investigation (p < 0.05).

**Table 4:** Association of L/A ratio between survivors and non-survivors (N = 100) The association was carried out using the Pearson chi-square test, and the p-value was significant at less than 0.05. L/A: lactate/albumin

Parameter	Outcome (mean ± SD)		p-value
	Survivors	Non-survivors	
L/A ratio (0 hour)	0.36 ± 0.27	1.63 ±1.11	0.003
L/A ratio (24 hours)	0.35 ± 0.23	0.88 ± 0.56	0.006
L/A ratio (48 hours)	0.33 ± 0.22	0.89 ± 0.55	0.002
L/A ratio (day 7)	0.30 ± 0.15	1.07 ± 0.47	0.000
L/A ratio (day 28)	0.21 ± 0.10	2.09 ± 0.02	0.018

Comparison of SOFA score between survivors and non-survivors

At admission, the mean SOFA scores for survivors and non-survivors in the study population were 4.71 and 10.09, respectively. This significant difference reflects a notably higher mean SOFA score in the group of patients who did not survive. As indicated in Table 5, the difference in the mean SOFA scores between survivors and non-survivors was statistically significant (p < 0.05) throughout the study period.

**Table 5:** Association of SOFA score between survivors and non-survivors (N = 100) The association was carried out using the Pearson chi-square test, and the p-value was significant at less than 0.05. SOFA: Sequential Organ Failure Assessment

Parameter	Outcome (mean ± SD)		p-value
	Survivors	Non-survivors	
SOFA score (0 hour)	4.71 ± 2.13	10.09 ± 3.48	0.000
SOFA score (24 hours)	4.46 ± 1.68	10.86 ± 2.88	0.000
SOFA score (48 hours)	4.77 ± 1.60	11.64 ± 2.40	0.000
SOFA score (day 7)	4.08 ± 1.08	12.20 ± 2.70	0.000
SOFA score (day 28)	3.61 ± 1.03	15.50 ± 0.71	0.001

Correlation of the L/A ratio with SOFA score

The correlation between the L/A ratio and SOFA score was assessed and found to be positive, as shown in Table 6. This positive correlation suggests that as the SOFA score increases, the L/A ratio also increases. The correlation was statistically significant (p < 0.05), indicating a less than 5% chance that this correlation is due to random variation in the sample.

**Table 6:** Correlation of the L/A ratio with SOFA score (N = 100) The correlation was performed using the Pearson chi-square test, and the p-value was significant at less than 0.05. L/A: lactate/albumin; r: Pearson correlation coefficient; SOFA: Sequential Organ Failure Assessment

SOFA score	L/A ratio	
	Pearson correlation (r)	p-value
0 hour	0.531	0.000
24 hours	0.327	0.001
48 hours	0.471	0.000
Day 7	0.692	0.000
Day 28	0.948	0.000

Association of septic shock and vasopressor requirement with outcome

The association between the presence or absence of septic shock and the requirement of vasopressors with outcome was observed. Among the 22 patients who died, 17 had septic shock and required vasopressor support within the first 24 hours to maintain their blood pressure. This suggests high mortality among those requiring vasopressor support in the first 24 hours, which was statistically significant (p < 0.05) (Table 7).

**Table 7:** Association between the requirement of vasopressors and the presence or absence of septic shock with outcome (N = 100) The association was carried out using the Pearson chi-square test, and the p-value was significant at less than 0.05.

Parameters		Outcome		Total	p-value
		Non-survivors	Survivors		
Vasopressors	No	5	67	72	0.000
	Yes	17	11	28	
Septic shock	Absent	5	58	63	0.000
	Present	17	20	37	
Total		22	78	100	-

Comparison of L/A ratio with serum lactate

**Figure 1:**
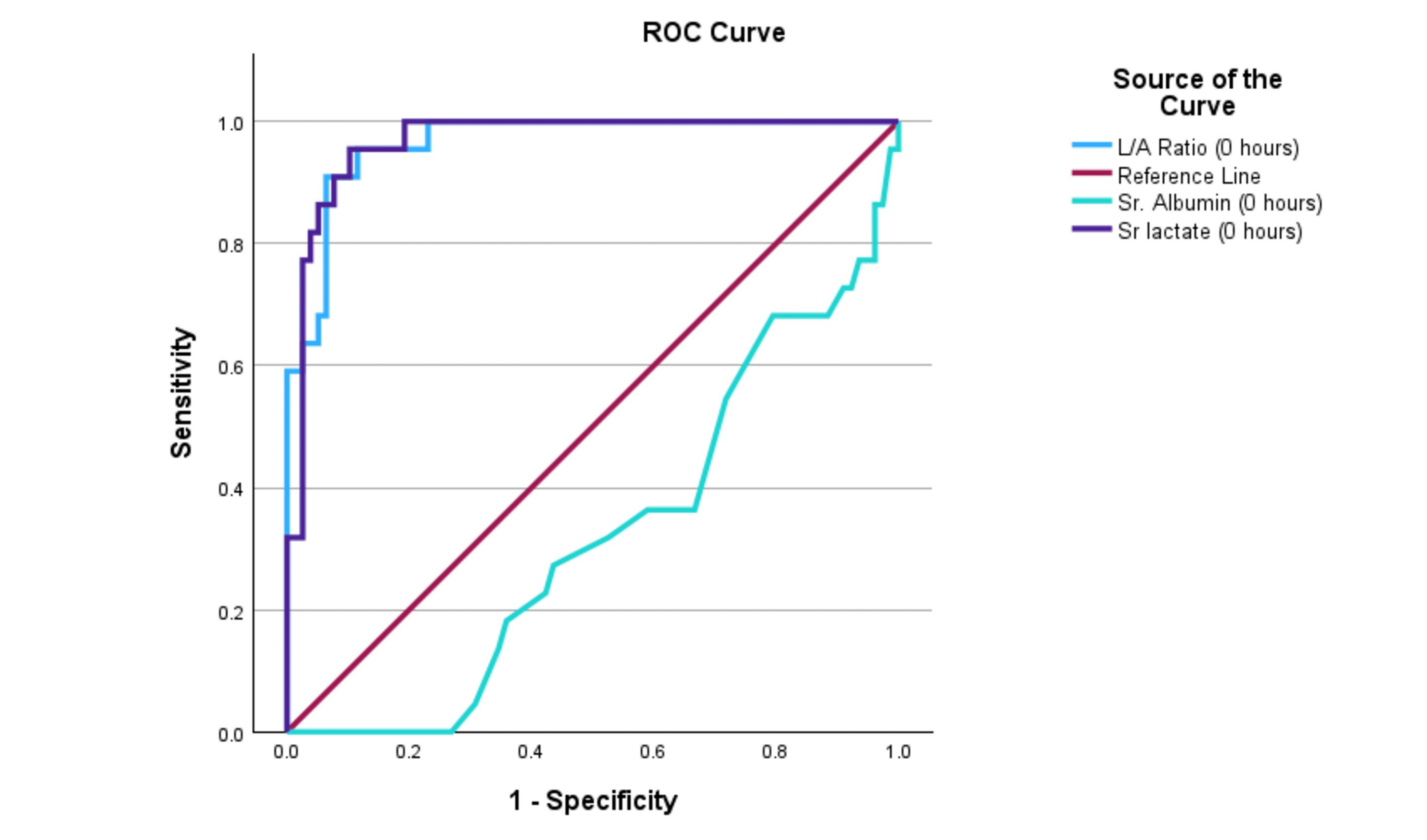
The AUROC shows a comparison among serum albumin, serum lactate, and L/A ratio on admission in patients with sepsis and septic shock. AUROC: area under the receiver operating characteristics; L/A: lactate/albumin; ROC: receiver operating characteristic

**Figure 2:**
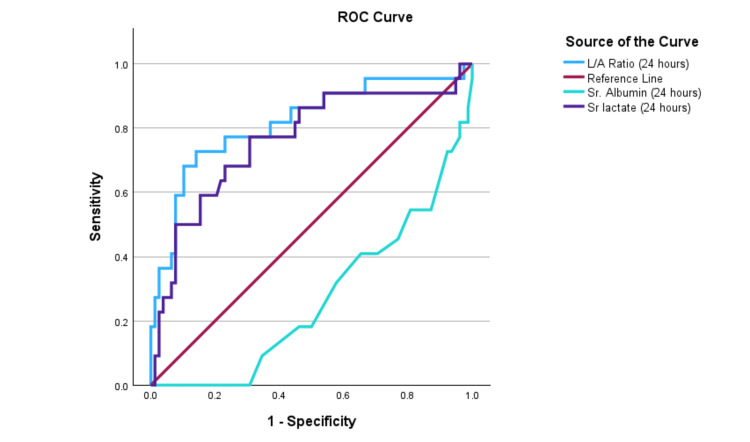
The AUROC shows a comparison among serum albumin, serum lactate, and L/A ratio at 24 hours in patients with sepsis and septic shock. AUROC: area under the receiver operating characteristics; L/A: lactate/albumin; ROC: receiver operating characteristic

**Figure 3:**
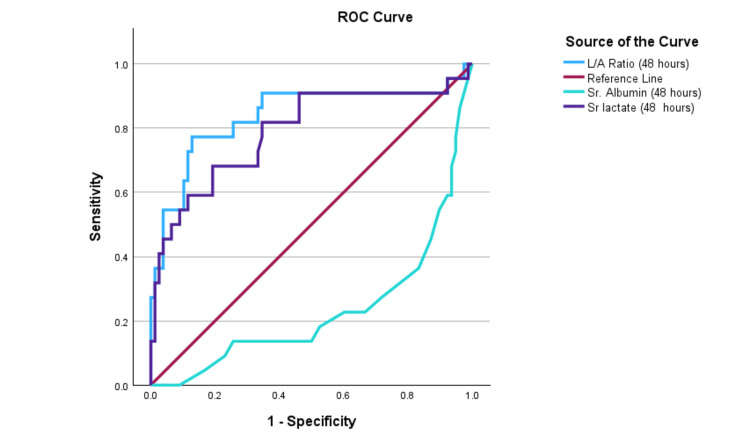
The AUROC shows a comparison among serum albumin, serum lactate, and L/A ratio at 48 hours in patients with sepsis and septic shock. AUROC: area under the receiver operating characteristics; L/A: lactate/albumin; ROC: receiver operating characteristic

The area under the receiver operating characteristic (AUROC) curve for serum albumin, serum lactate, and L/A ratio was obtained. The area under the curve (AUC) values for serum lactate and L/A ratio at 24 hours were 0.76 and 0.81, respectively, with a cutoff for L/A ratio of 0.60. At 48 hours, the AUC values for serum lactate and L/A ratio were 0.78 and 0.83, respectively, as shown in Table 8. Diagrammatic representations of the AUROC at admission, 24 hours, and 48 hours are shown in Figure 1, Figure 2, and Figure 3, respectively. The ROC curves indicate that the L/A ratio (blue) consistently exhibits the best diagnostic performance at 0, 24, and 48 hours, with sensitivity and specificity nearing 95%, 92%, and 92%, respectively. Serum lactate (purple) also shows high diagnostic accuracy, closely following the L/A ratio with near-perfect sensitivity and specificity. Overall, the L/A ratio proves to be the most reliable indicator, followed by serum lactate, while serum albumin is the least effective.

**Table 8:** The AUROC value of serum albumin, serum lactate, and L/A ratio in patients with sepsis and septic shock on admission at 24 hours and 48 hours AUROC: area under the receiver operating characteristics; AUC: area under the curve; L/A: lactate/albumin

Test result variable(s)	AUC values
L/A ratio of (0 hour)	0.966
Serum lactate (0 hour)	0.967
Serum albumin (0 hour)	0.323
L/A ratio (24 hours)	0.818
Serum lactate (24 hours)	0.762
Serum albumin (24 hours)	0.268
L/A ratio (48 hours)	0.837
Serum lactate (48 hours)	0.789
Serum albumin (48 hours)	0.232

## Discussion

Sepsis is a serious medical illness wherein the body's immune reaction to an infectious agent can result in extensive inflammation, which, if left untreated, can cause tissue destruction, multiple organ failure, and even death. Sepsis occurs as a result of the complex interplay between pro-inflammatory and anti-inflammatory responses. Initially, sepsis triggers a robust pro-inflammatory response aimed at combating the infection. This phase involves the release of a cascade of mediators such as IL-6, TNF-α, and IL-1, along with the activation of various immune cells, leading to systemic inflammation. This response, while crucial for pathogen clearance, can cause collateral tissue damage and organ dysfunction if not properly regulated. Following the hyper-inflammatory phase, many patients enter a phase of immunosuppression characterized by apoptosis of immune cells, reduced production of pro-inflammatory cytokines, and elevated levels of anti-inflammatory cytokines such as IL-10 [10].

Serum lactate is a well-known marker of sepsis. However, many studies have demonstrated that serum lactate can be affected by various factors. Medications, such as salbutamol and metformin, can significantly elevate serum lactate levels in the absence of infection [11,12]. Additionally, lactate clearance can be impaired in the presence of liver disease [13]. Some studies have shown low to normal levels of serum lactate even in the presence of severe infections [14]. These limitations have prompted the inclusion of more robust markers such as the L/A ratio.

In our study, the non-survivor group had higher levels of serum lactate than the survivors, although the difference was not statistically significant. Several studies in the past also indicate the superiority of the L/A ratio compared to serum lactate levels alone. One such study showed a higher significance of the L/A ratio compared to serum lactate levels alone in patients with sepsis [15]. In our study, the L/A ratio was significantly higher among the non-survivor population on admission, at 24 hours, 48 hours, day 7, and day 28 compared to survivors, and these differences were statistically significant (p < 0.05). Shin et al. investigated the relevance of the L/A ratio in 946 cases using information from a multi-center registry involving ten emergency departments [16]. In their study, the median L/A ratio was 1.7 in the non-survivor group, compared to one in the survivor group. The results of our study, demonstrating that the L/A ratio had superior predictive value in patients with sepsis and septic shock, were further supported by this study.

A study was conducted to evaluate the usefulness of the L/A ratio in risk classification for individuals with sepsis admitted to the ICU. Similar outcomes were observed in this study, which compared case-controls with an L/A ratio < 0.15 adjusted for Acute Physiology and Chronic Health Evaluation 2 (APACHE-2) values to 99 patients with an L/A ratio > 0.15. In paired analysis, a 27% difference in in-hospital outcomes was found between the group with an L/A ratio > 0.15 and the group without one [17]. The AUROC curves were obtained for serum lactate, serum albumin, and L/A ratio. The AUC value for serum lactate and L/A ratio was almost the same at admission, while they were significantly higher at 24 hours and 48 hours. Based on the ROC curve, the cutoff for the L/A ratio at 24 hours was determined to be 0.50. These results were similar to those of a prospective study conducted by Bou Chebl et al. [18], suggesting that the L/A ratio has a higher predictive value than serum lactate levels alone. In addition, compared to survivors, the mortality group had much higher needs for vasopressors and mechanical ventilation within the first 24 hours. Similar results were found in a study indicating poor prognosis in those who require vasopressors and mechanical ventilation with a high L/A ratio at the time of admission and thereafter [18].

Organ dysfunction is assessed early in the course of the disease using the SOFA score, which helps identify the severity of the disease and allows prompt management. In our study, the mean value of the SOFA score upon arrival in survivors was 4.71, compared to 10.09 in those who died. Similarly, the mean value of the SOFA score on day 7 was 4.08 in the survivor group, while it was 12.20 in the mortality group. In agreement with our study, Liu et al. conducted a study on the SOFA score and its clinical implications in sepsis. They reported similar results to our study, with the mean SOFA score in survivors being 7.85 and 12.05 in the mortality group [19]. The correlation of the novel marker of sepsis, like the L/A ratio, was performed with the already established marker, the SOFA score. The Pearson correlation coefficient was 0.531 at admission, 0.327 at 24 hours, 0.471 at 48 hours, 0.692 at day 7, and 0.948 at day 28, all of which were statistically significant (p < 0.01). Similar findings were obtained in another study by Purohit et al., which showed a correlation coefficient of 0.828 and was also statistically significant [20].

In our study, septic shock was present in 37 patients, of whom 17 died. The association between the presence of septic shock and outcome was assessed, and it was found to be statistically significant (p < 0.05). This suggests that the presence of septic shock is associated with a poor prognosis. This result is further supported by a study conducted at the University of Pittsburgh Medical Center and Kaiser Permanente Northern California, which showed mortality rates of 54% and 35% among patients with septic shock, respectively [1].

Limitations

One limitation of our study is the relatively small sample size, which may not be representative of the larger population. Additionally, the study's single-center design could limit the generalizability of the findings to other settings. The observational, cross-sectional analysis introduces potential biases, and the heterogeneity of the patient population may affect the results. Confounding factors, such as medication use and comorbidities, could influence serum lactate and albumin levels, impacting the L/A ratio. The lack of long-term follow-up data further limits the study. Future research with larger, multi-center, prospective designs and standardized protocols is necessary.

## Conclusions

Our study demonstrated a strong positive correlation between the L/A ratio and SOFA score in individuals with sepsis, which is linked to poor prognosis and high mortality rates. A high L/A ratio at the time of admission can act as an independent indicator of ICU admission, in addition to the SOFA score. The L/A ratio is potentially useful for assessing sepsis in critically ill patients admitted to the ICU, as well as for early detection and predicting unfavorable outcomes. Our research clearly shows that the L/A ratio significantly improves predictive performance compared to conventional serum lactate alone. Timely management of sepsis not only improves outcomes but also reduces some of the permanent damage that follows sepsis, such as kidney injury.

## References

[1] Singer M, Deutschman CS, Seymour CW (2016). The third international consensus definitions for sepsis and septic shock (Sepsis-3). JAMA.

[2] Sprung CL, Peduzzi PN, Shatney CH, Schein RM, Wilson MF, Sheagren JN, Hinshaw LB (1990). Impact of encephalopathy on mortality in the sepsis syndrome. Crit Care Med.

[3] Fleischmann C, Scherag A, Adhikari NK (2016). Assessment of global incidence and mortality of hospital-treated sepsis. Current estimates and limitations. Am J Respir Crit Care Med.

[4] Angus DC, van der Poll T (2013). Severe sepsis and septic shock. N Engl J Med.

[5] Vincent JL, Moreno R, Takala J (1996). The SOFA (Sepsis-related Organ Failure Assessment) score to describe organ dysfunction/failure. Intensive Care Med.

[6] Shankar-Hari M, Phillips GS, Levy ML (2016). Developing a new definition and assessing new clinical criteria for septic shock. JAMA.

[7] Vincent JL, de Mendonça A, Cantraine F (1998). Use of the SOFA score to assess the incidence of organ dysfunction/failure in intensive care units: results of a multicenter, prospective study. Crit Care Med.

[8] Kruse O, Grunnet N, Barfod C (2011). Blood lactate as a predictor for in-hospital mortality in patients admitted acutely to hospital: a systematic review. Scand J Trauma Resusc Emerg Med.

[9] Chatterjee S, Bhattacharya M, Todi SK (2017). Epidemiology of adult-population sepsis in India: a single center 5 year experience. Indian J Crit Care Med.

[10] Reinhart K, Bauer M, Riedemann NC, Hartog CS (2012). New approaches to sepsis: molecular diagnostics and biomarkers. Clin Microbiol Rev.

[11] Smith ZR, Horng M, Rech MA (2019). Medication‐induced hyperlactatemia and lactic acidosis: a systematic review of the literature. Pharmacotherapy.

[12] Misbin RI, Green L, Stadel BV, Gueriguian JL, Gubbi A, Fleming GA (1998). Lactic acidosis in patients with diabetes treated with metformin. N Engl J Med.

[13] Sterling SA, Puskarich MA, Jones AE (2015). The effect of liver disease on lactate normalization in severe sepsis and septic shock: a cohort study. Clin Exp Emerg Med.

[14] Bou Chebl R, Jamali S, Sabra M (2020). Lactate/albumin ratio as a predictor of in-hospital mortality in septic patients presenting to the emergency department. Front Med (Lausanne).

[15] Choi SJ, Ha EJ, Jhang WK, Park SJ (2021). Association between the lactate/albumin ratio and mortality in pediatric septic shock patients with underlying chronic disease: retrospective pilot study. Minerva Pediatr (Torino).

[16] Shin J, Hwang SY, Jo IJ (2018). Prognostic value of the lactate/albumin ratio for predicting 28-day mortality in critically ill sepsis patients. Shock.

[17] Lichtenauer M, Wernly B, Ohnewein B (2017). The lactate/albumin ratio: a valuable tool for risk stratification in septic patients admitted to ICU. Int J Mol Sci.

[18] Bou Chebl R, Geha M, Assaf M (2021). The prognostic value of the lactate/albumin ratio for predicting mortality in septic patients presenting to the emergency department: a prospective study. Ann Med.

[19] Liu C, Suo S, Luo L, Chen X, Ling C, Cao S (2022). SOFA score in relation to sepsis: clinical implications in diagnosis, treatment, and prognostic assessment. Comput Math Methods Med.

[20] Purohit A, Yadav A, Yadav R, Kumar S, Prasad S, Gupta D (2024). Lactate albumin ratio as predictor of 28 days mortality in septic shock patients and its correlation to SOFA score for assessment of organ dysfunction: a prospective observational study. Arch Anesth Crit Care.

